# COVID-19-Associated Parosmia and Dysgeusia: A Case Series

**DOI:** 10.7759/cureus.17584

**Published:** 2021-08-30

**Authors:** Javier A Ivona, Yonathan Cortés Vega

**Affiliations:** 1 Emergency, Sanatorio Juan XXIII, General Roca, ARG; 2 Primary Care, Pueblo Seco Rural Health Clinic, San Ignacio, CHL

**Keywords:** covid-19, hyposmia, anosmia, hypogeusia, ageusia, parosmia, dysgeusia, chemical senses, chemosensory dysfunction, loss of smell

## Abstract

Impairment of the chemical senses - smell, taste, and chemesthesis - has been pinpointed as one of the main clinical presentations of coronavirus disease 2019 (COVID-19). Chemosensory dysfunction can be quantitative, involving reduction or loss of perception (e.g., hyposmia, anosmia, hypogeusia), and qualitative, involving distortion of perception (parosmia and dysgeusia). Quantitative chemosensory dysfunction is reported more often among COVID-19 patients than qualitative dysfunction. The following report details four patients with a laboratory-assisted diagnosis of COVID-19 who experienced qualitative chemosensory dysfunction. A discussion of these symptoms in the broader context of upper respiratory tract infections is included, with an emphasis on olfactory dysfunction.

## Introduction

Chemosensory perception is mediated in humans by three distinct modalities: smell, taste, and chemesthesis. The specialized olfactory epithelium (OE) on the roof of the nasal cavity interfaces with volatile chemicals, also known as odorants. Output signals from the OE advance along the olfactory nerve and reach the olfactory bulb and other parts of the central nervous system (CNS). The perception of smell begins to take place when odorants reach the OE using one of two possible entry routes. Orthonasal olfaction is the sensing of outside odorants that enter the nasal cavity through the nostrils; retronasal olfaction is the sensing of odorants within the oral cavity that reach the OE via the nasopharynx. It has been estimated that the number of distinct olfactory stimuli that can be perceived by humans is on the order of one trillion [1]. Non-volatile chemicals, also known as tastants, are sensed when they interact with taste receptors located on gustatory cells. The gustatory cells are embedded in the tongue and epiglottis and their output is transmitted to the CNS via the facial, the glossopharyngeal, and the vagus nerves. The perception of taste is thus a consequence of the activation of any combination of sweet, salty, sour, bitter, and umami taste receptors. Evidence is accumulating that suggests the existence of a further receptor for fat taste [2]. Chemesthetic sensations arise when non-volatile compounds activate pain, touch, and temperature receptors within the nose and mouth, triggering signals that are relayed by the trigeminal nerve. The perception of flavor is a product of CNS integration of input from all three receptor types, olfactory, gustatory, and chemesthetic, which may contribute unequal shares of the sensory experience. Certain flavors are shaped primarily by their smell dimension: apples and pears have near-identical flavors if retronasal olfaction is suppressed. Other flavors are drawn mainly from the chemesthetic input. Examples of this are the coolness of mints, which is mediated by menthol, and the tingling sensation of soft drinks containing carbon dioxide.

The ability of *Coronaviridae* to disrupt chemosensory function had been noted following the 2002 severe acute respiratory syndrome coronavirus (SARS-CoV) outbreak and, in February of 2020, the first reports of hyposmia among SARS coronavirus 2 (SARS-CoV-2)-infected patients were published by researchers from Wuhan [3,4]. In recent months, hyposmia and hypogeusia have been increasingly recognized as habitual symptoms of coronavirus disease 2019 (COVID-19). While not the chief cause of mortality in patients with the disease, impairment of chemosensory function can be harmful. Olfactory dysfunction might diminish the ability of individuals to detect environmental hazards such as combustion or spoiled food. Olfactory as well as gustatory dysfunction can aid unintentional changes in appetite and weight and be associated with depressive symptoms [5,6].

The present report details four patients that experienced qualitative impairment of olfactory or gustatory function.

## Case presentation

Patient 1

A 25-year-old woman with an unremarkable past medical history (PMH) underwent drive-through testing for COVID-19 after experiencing low-grade fever, muscle pain, a mild headache, and hyposmia. The nasal swab sample was collected on day 6 after symptom onset, and reverse transcription-polymerase chain reaction (RT-PCR) was positive for SARS-CoV-2. The patient did not seek medical attention or self-medicate. On day 10 after symptom onset, her symptoms had cleared, with the exception of hyposmia that lasted until day 14 before resolving fully.

The woman went on to visit a health clinic nine weeks after recovering from this episode, at which time she reported a perturbation to her sense of taste. Her chief complaint was that cola-flavored soft drinks did not feel the same as she remembered. She likened the flavor of certain carbonated sodas to that of “drinking perfume” and “tasting rubbing alcohol”. The patient was unable to pinpoint the day she had started to notice these symptoms. The flavor of other beverages, including non-cola-flavored sodas, would feel the same way as she remembered. The patient had not become aware of any disturbances in chemesthetic or olfactory function. She was referred to an otolaryngologist for specialist management.

Patient 2

A 17-year-old boy with an unremarkable PMH developed pharyngodynia, muscle pain, and headaches. His symptoms worsened over the following days and he would also experience colicky abdominal pain, vomiting, hyposmia, and hypogeusia. This prompted him to consult a physician over the phone on day 11 after symptom onset. It was advised to the patient that he undergo RT-PCR testing, which resulted in the detection of SARS-CoV-2 in a nasal swab sample. He was prescribed 500 mg acetaminophen PO three times a day (t.i.d.), 100 mg aspirin PO as needed, and 0.5/40 mg atropine/papaverine PO as needed, which contributed to alleviating the symptoms. On day 18 after symptom onset, his condition had resolved fully.

Two months after this episode, the patient presented to a primary care clinic complaining of altered smell perception. Whenever the patient was within range of odorants coming from cooked chicken, hot milk, raw asparagus, raw broccoli, raw celery, cooked pasta, or mustard sauce, he would feel an “unfamiliar" smell that was accompanied by a “perturbing” sensation. This would often be followed by nausea and vomiting. These symptoms had resulted in decreased appetite and reduced food intake. The patient was prescribed 10 mg domperidone PO t.i.d. to alleviate nausea and showed some improvement. He began to attend check-up appointments every two weeks from then onwards and, over the first three months, reported an unintentional body weight loss of 3 kg (6.6 lbs). Chemosensory function was noted to be recovering between visits; his appetite, body weight, and chemosensory function returned to baseline six months after his first follow-up appointment.

Patient 3

The patient was a 67-year-old man with a PMH of 3-pack-year smoking, hypertension, and hyperlipidemia. The patient developed progressive, nonproductive cough over a period of several weeks. During this time, he noticed a decrease in his appetite along with unintentional body weight loss of 12 kg (26.4 lbs). The patient’s nonproductive cough became productive of blood-stained mucus after five months, which led him to visit our emergency department. On physical examination, his vital signs were within the healthy range; his oxygenation status was reassuring. He was admitted for workup of his symptoms, tested for SARS-CoV-2, and placed under contact and droplet precautions in accordance with the hospital protocol; 20 mg omeprazole PO q.d. and 40 mg enoxaparin subcutaneous q.d. were added to his medication regimen upon admission. A positive RT-PCR result and an unrevealing CT scan of his chest enabled the diagnosis of mild COVID-19.

The patient began experiencing parosmia 14 days after admission: when attempting to eat a variety of meals he would report a nauseating, “oily” smell that was most potently evoked by cooked chicken. He began to eat primarily bread and water biscuits and to refuse consumption of other foods. He reported a further reduction of his appetite and decreased frequency of bowel movements coinciding with the start of parosmic symptoms.

His clinical course was subsequently uncomplicated save for persistent hyposmia. A second RT-PCR test performed on day 19 from admission was negative, and the patient was discharged home on day 21.

Patient 4

**Figure 1:**
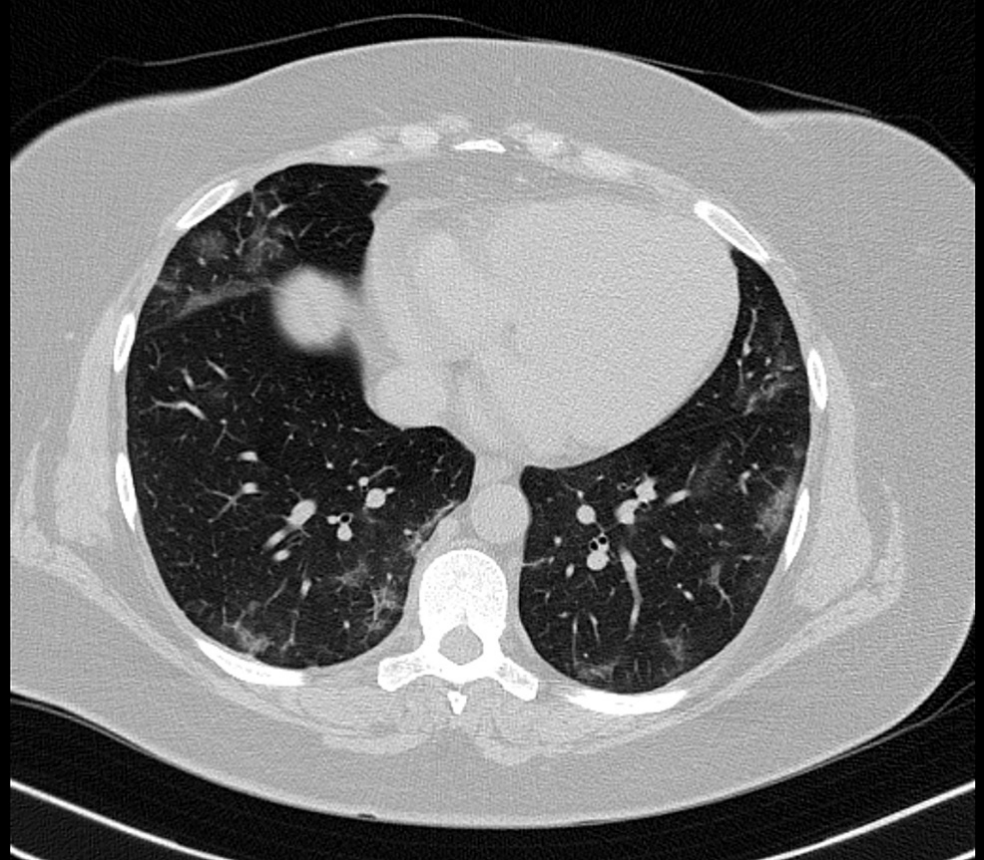
Non-contrast chest CT scan Multifocal, bilateral areas of ground-glass opacification are noted. Lesions were found predominantly in the inferior lobes. The percentage of lung involvement was approximately 5% by visual assessment.

A 47-year-old woman arrived at the emergency department five days after the onset of nonproductive cough, joint and muscle pain, vomiting, watery diarrhea, and loss of taste. Her PMH was remarkable for class I obesity and a 10-pack-year smoking history. The patient was a poor historian and, on a second telling of her symptoms, she acknowledged taste distortion rather than taste loss. She noticed while eating a salad that its flavor was unusual and would trigger a nauseating feeling. When asked to further characterize the flavor, she described it as “oily” and said that it was decreasing her appetite. On physical examination, the patient had stable vital signs, a normal reading on pulse oximeter, and was in no respiratory distress. During auscultation of the chest, bibasilar crackles were noted; a subsequent CT scan revealed findings suggestive of viral pneumonia (Figure 1). A nasal swab RT-PCR was positive for SARS-CoV-2, and a diagnosis was made of moderate COVID-19. The patient was instructed to remain isolated at her home. She was followed up over the phone at two-day intervals and was discharged on day 16 after symptom onset, having made a full recovery.

## Discussion

Olfactory dysfunction is a well-known complication of several upper respiratory tract infections (URTIs) such as influenza, rhinovirus infection, and infection by human parainfluenza viruses. These agents can cause hyposmia in the absence of rhinitis and nasal obstruction; however, hyposmia is usually a result of nasal obstruction and presents along with it in most cases [7]. In contrast, COVID-19-related hyposmia is typically associated neither with rhinorrhea nor with nasal obstruction; it is also more frequent with COVID-19 than it is with influenza [8,9]. The incidence of hyposmia among COVID-19 patients can be gleaned from the literature: a study of 417 mild-to-moderate patients from Europe reported hyposmia or anosmia in 85% of cases [8]. A study of 68 patients from France reported olfactory dysfunction in 75% of cases [10]. It is noteworthy that self-reported rates of hyposmia are often considerably lower than those obtained from objective assessments of olfactory function, suggesting that hyposmia goes unnoticed by many patients [11]. Furthermore, it can be challenging for an individual experiencing altered flavor perception to accurately identify the sensory modality responsible for the change. Although the reported figures were similar for the incidence of olfactory and gustatory dysfunction in these two studies, it should be taken into account that loss of retronasal olfaction can be mistakenly reported as loss of taste. This raises the possibility that the incidence of true gustatory dysfunction is lower [12]. Qualitative chemosensory dysfunction has been reported less frequently than hyposmia or hypogeusia. When the incidence of parosmia or dysgeusia is calculated from self-reports, it is possible to underestimate it given that patients can remain unaware of these symptoms or underestimate their importance. Qualitative changes in chemosensory function ought to be actively inquired about in order to avoid misdiagnosing them. In our case sample, Patient 3 initially interpreted his parosmic symptoms as hyposmia, and only seemed to become aware of a qualitative dysfunction when asked whether the food felt any different from what he remembered. Patient 4 also appeared to mistake dysgeusia with hypogeusia, and only admitted to a qualitative change in perception when this was asked about specifically. Patients 2, 3, and 4 reported nausea, food aversion, and a decreased appetite. This can be interpreted to suggest that the risk for weight loss, as experienced by Patient 2, is higher with qualitative than quantitative chemosensory dysfunction. Based on the results of a questionnaire administered to 4039 individuals with either a clinical or laboratory-assisted diagnosis of COVID-19, parosmia was present in about 7% of cases [12]. Parosmia usually presents along with hyposmia or anosmia. Parosmic sensations are usually unpleasant and are often described as “rotten”, “fecal”, or “foul”. The prevalence of parosmia in the general population has been previously estimated at about 4%; a significant share of affected individuals may, however, be aware only of a quantitative dysfunction [13]. In a study of 56 patients with parosmia from France, the commonest clinical associations were URTI, nasal and paranasal sinus disease, and head trauma [14]. The majority of patients with parosmia will show spontaneous, gradual improvement; the usual treatment is watchful waiting [15].

There are two proposed mechanisms to explain URTI-associated olfactory dysfunction. The central mechanism hypothesis posits that the virus gains access to areas of the brain that mediate olfactory function (e.g., the medial temporal lobe, the insula, or the orbitofrontal cortex). This takes place through hematogenous spread or retrograde neuronal transport involving the olfactory and trigeminal nerves. Once the virus penetrates the CNS, it replicates and then triggers an inflammatory response that interferes with olfactory function. Such neuroinvasive capabilities have been thoroughly characterized for other members of the *Betacoronavirus* genus such as the mouse hepatitis virus (MHV), and evidence is accruing that suggests a similar potential for human-infecting coronaviruses [16]. The peripheral mechanism hypothesis states that olfactory dysfunction is the result of virus-induced damage of the OE. Under this hypothesis, hyposmia can be explained by the interruption of output from olfactory sensory neurons (OSN) in the injured OE. Attempts at explaining parosmia warrant a cursory review of physiology. An OSN expresses only one of around one thousand different receptor types. Each receptor type has varying levels of affinity to multiple odorants. An OSN synapses with a single glomerulus in the olfactory bulb, but the glomerulus receives input from multiple OSNs that all express the same receptor type. When an odorant comes into contact with the OE, it binds multiple OSNs with different receptor types. Since different receptor types relay to different glomeruli, a specific excitation pattern is triggered in the olfactory bulb encoding information about the odorant. When the OE is damaged, the differential impairment of OSNs expressing different receptor types alters downstream excitation patterns and results in a defective characterization of the odorant [17]. It is interesting to note that both olfactory and gustatory symptoms are reported more frequently in women than men, more frequently in the young than the old, and appear to be rare in Asian cohorts [8,9]. A 2020 review encompassing 1556 Chinese COVID-19 patients reported chemosensory dysfunction in none of its subjects [18].

## Conclusions

The presence of chemosensory dysfunction appears to have more sensitivity for COVID-19 than for other URTIs. Chemosensory dysfunction can go unnoticed by patients and its incidence can be underestimated by clinicians. COVID-19-associated parosmia and dysgeusia, while less frequent, are potentially more harmful than anosmia or ageusia because the former can be associated with food aversion and loss of weight.
